# Supplementary material for: Causal Relationship between Aging and Anorexia Nervosa: A White-Matter-Microstructure-Mediated Mendelian Randomization Analysis
Source: Biomedicines. 2024 Aug 16;12(8):1874. doi: 10.3390/biomedicines12081874 (PMC11351342; doi:10.3390/biomedicines12081874)
Supplement: Supplementary file 1 [file biomedicines-12-01874-s001.zip › Supplementary Figure/Figure S7/Single SNP plot of MR analysis for ubm-b-1975 to AN.pdf]

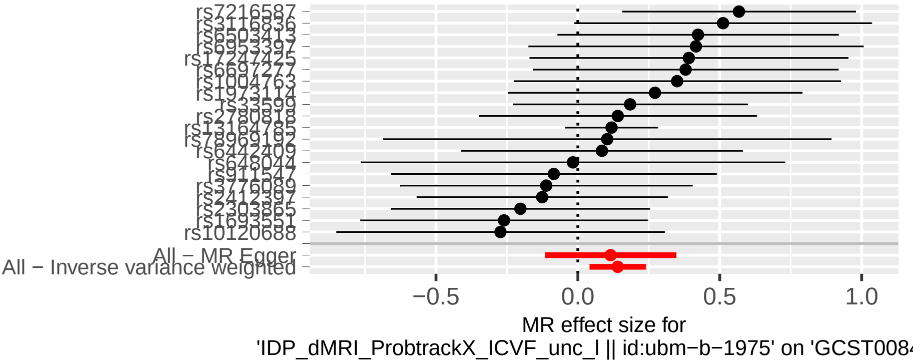
rs7216587
rs3116836
rs6503413
rs6953397
rs17247425
rs6697277
rs1004763
rs1973114
rs33599
rs2780818
rs13164785
rs78969192
rs6442409
rs648044
rs911547
rs3776089
rs2412397
rs2303865
rs1693551
rs10120688
All − MR Egger
All − Inverse variance weighted
−0.5
0.0
0.5
1.0
MR effect size for
'IDP_dMRI_ProbtrackX_ICVF_unc_l || id:ubm−b−1975' on 'GCST008
